# Monocyte-to-high-density lipoprotein ratio and systemic inflammation response index are associated with the risk of metabolic disorders and cardiovascular diseases in general rural population

**DOI:** 10.3389/fendo.2022.944991

**Published:** 2022-09-09

**Authors:** Pengbo Wang, Xiaofan Guo, Ying Zhou, Zhao Li, Shasha Yu, Yingxian Sun, Yu Hua

**Affiliations:** The Department of Cardiology, The First Hospital of China Medical University, Shenyang, China

**Keywords:** monocyte-to-high-density lipoprotein ratio, systemic inflammation response index, metabolic disorders, metabolic syndrome, 10-year cardiovascular disease risk

## Abstract

**Background:**

The present study aimed to clarify the effects of four inflammatory indicators (monocyte-to-high-density lipoprotein ratio [MHR], neutrophil-to-lymphocyte ratio [NLR], systematic immune-inflammation index [SII], and systemic inflammation response index [SIRI]) in evaluating the risk of metabolic diseases and cardiovascular disease (CVD), filling the gap of inflammation-metabolism system research in epidemiology.

**Methods:**

We conducted a cross-sectional study and multivariable logistic regression analysis to elucidate the association between inflammatory indicators and metabolic diseases and CVD risk. Metabolic diseases were defined as metabolic disorders (MetDs) or their components, such as metabolic syndrome (MetS), dyslipidemia, and central obesity. We calculated the Framingham risk score (FRS) to evaluate 10-year CVD risk.

**Results:**

Odds ratios for the third vs. the first tertile of MHR were 2.653 (95% confidence interval [CI], 2.142–3.286) for MetD, 2.091 (95% CI, 1.620–2.698) for MetS, 1.547 (95% CI, 1.287–1.859) for dyslipidemia, and 1.515 (95% CI, 1.389–1.652) for central obesity. Odds ratios for the third vs. the first tertile of SIRI were 2.092 (95% CI, 1.622–2.699) for MetD, 3.441 (95% CI, 2.917–4.058) for MetS, 1.417 (95% CI, 1.218–1.649) for dyslipidemia, and 2.080 (95% CI, 1.613–2.683) for central obesity. The odds ratio of a 10-year CVD risk of >30% for the third vs. the first tertile of MHR was 4.607 (95% CI, 2.648–8.017) and 3.397 (95% CI, 1.958–5.849) for SIRI.

**Conclusions:**

MHR and SIRI had a significant association with MetD and its components, in which a higher level of MHR or SIRI tended to accompany a higher risk of metabolic diseases. Furthermore, they also correlated with CVD, and the increment of these indicators caused a gradually evaluated risk of 10-year CVD risk.

## Introduction

Metabolic diseases have been recognized as crucial risk factors and chronic pathology processes in the elderly population, and increasing evidence has confirmed that metabolic dysfunction is the basis of various chronic diseases, such as diabetes mellitus (DM), and cardiovascular disease (CVD) events (1–3). Various studies have revealed that multiple metabolic diseases in China were prevalent, and the prevalence was gradually increasing (3), with the prevalence of 31.1% for metabolic syndrome (MetS) (4), 33.8% for dyslipidemia (5), and 40.8% for central obesity (4). The northeast rural regions of China even had a higher prevalence of MetS (37.3% for men and 45.8% for women) than the general level due to multiple chronic disease-related lifestyles, such as a high-salt diet (6), which causes a heavy burden of chronic diseases and CVD. Therefore, it is critical to elucidate the potential mechanism and associated risk factors for metabolic diseases and further explore the possible intervention strategies.

The metabolic and immune systems are among the essential requirements for the homeostasis of the human body (7). Increasing evidence has shown a high-grade inflammatory response in adipose tissue with a prevalent infiltration of macrophages and other immune cells among the adipocytes, during which cells changed from anti-inflammatory into pro-inflammatory status under this infiltration of immune cells, suggesting that chronic inflammation could potentially be related to obesity (8). Additionally, cellular metabolic processes could also be regulated by inflammatory molecules or inflammatory pathways, such as cytokines (tumor necrosis factor [TNF]-α, interleukin [IL]-6, and IL-1β), which could act in an autocrine or paracrine manner and interfere with the insulin signaling in peripheral tissues by activating the c-Jun N-terminal kinase (JNK) pathway or nuclear factor kappa B (NF-κB) pathway, inducing β-cell dysfunction and promoting insulin resistance (9, 10). In addition, multiple inflammatory factors are involved in the regulation of various metabolic products, such as lipids and glucose (11, 12), and high-grade inflammation could lead to extensive accumulation of abnormal lipids and glucose, eventually resulting in metabolic dysfunction.

Recent clinical research has indicated that salicylate sodium therapy or treatment with inflammatory cytokine inhibitors could significantly reduce the serum glucose of type 2 DM (T2DM) patients and decrease the risk of CVD events (12–14). However, most current studies focused on inflammation and metabolism have been conducted on adipose tissue or immune cells, and most diseases at the individual level have been limited to obesity and DM, representing only a limited aspect of metabolic diseases, especially in the elderly population in whom obesity is not a typical metabolic alteration, leading to a limitation of epidemiological studies on inflammation and metabolic disorders. Moreover, cellular metabolic dysfunction or abnormal metabolism of cellular products could be restored by multiple homeostatic mechanisms and compensatory behaviors, which might only manifest as a pre-metabolic imbalance status at the individual level and, thus, could not cause the symptoms of a metabolic disorder (MetD). Furthermore, previous studies have usually used inflammatory factors, such as IL-1/6 and TNFα, to reflect inflammation levels, which seemed more suitable for molecular research. Additionally, in clinical or large-scale population screening, the blood cell count has been more commonly measured to reflect the inflammatory status.

The composite inflammatory indicators such as monocyte-to-high-density lipoprotein ratio (MHR) (15), neutrophil-to-lymphocyte ratio (NLR) (15), systematic immune-inflammation index (SII) (16), and systemic inflammation response index (SIRI) (17) are a novel type of parameters based on the traditional peripheral blood cell count, calculated by combining different biochemical parameters to balance inflammation and immunity status (17). Previous studies have confirmed that these indicators could reflect inflammation levels and were widely used in evaluating the risk of various chronic diseases and the prognosis of tumors (18–22). Therefore, our study enrolled and screened these four simple-to-calculate and easily accessible systemic inflammatory indicators to assess their effect in evaluating the risk of metabolic diseases and CVD, filling the knowledge gap on the association between inflammation and metabolic diseases at the individual level.

## Methods

### Study population

**Figure 1:**
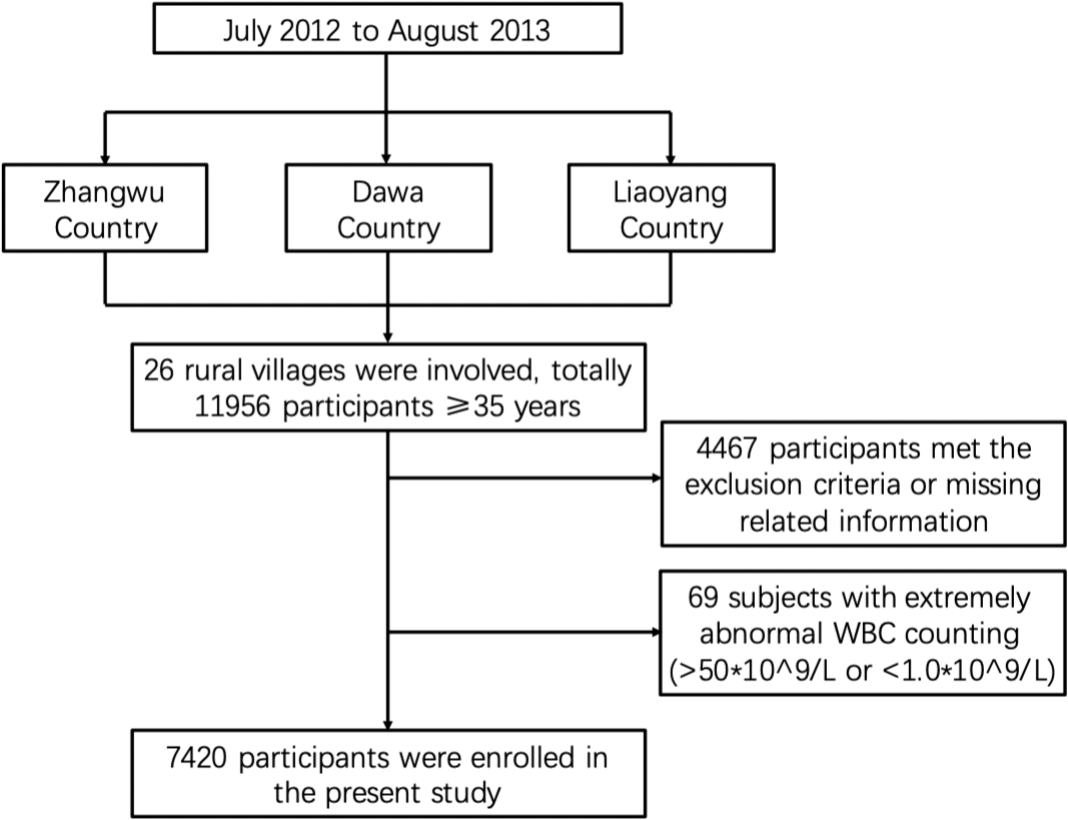
Flowchart of our selected study population protocol. We randomly selected 26 villages from three countries in northeastern China from July 2012 to August 2013. In total, 11,956 participants were enrolled in our study. After excluding people who did not meet the research criteria, such as those with cancer, pregnancy, and missing related information, and further excluding 69 subjects with extremely abnormal WBC counts, we finally got a study population of 7,420 subjects.

The present study was based on the Northeast China Rural Cardiovascular Health Study (NCRCHS), aiming to further reveal the association between various inflammatory indicators and the risk of metabolic diseases. We conducted the baseline study from July 2012 to August 2013, and the detailed protocol was described in previous research (23, 24), which is summarized in **Figure 1**. In brief, we recruited 11,956 residents of rural regions (aged ≥35 years) from 26 villages in three countries in Northeastern China. However, there were 4,467 subjects who met the exclusion criteria, which included pregnancy, cancer, mental disorders, or failure to complete related research, such as those with incomplete data. We further excluded 69 subjects with extremely abnormal white blood cell (WBC) counts (>50 × 10^9^/l or <1.0 × 10^9^/l) to avoid the effects of the acute phase of infection on the inflammatory status. Ultimately, we obtained a target population of 7,420 people.

### Data collection and ethics

We established a cardiologist team to conduct outpatient face-to-face interviews with participants and complete paper vision standard questionnaires to collect data. Before the project, we conducted a training course about project-related knowledge and ethical content. Only the staff who passed the related test could be authorized to conduct subsequent research.

The Ethics Committee of China Medical University approved our project (Shenyang, China, ethical approved project identification code: AF-SOP-07-1, 0-01). Every participant received and signed a paper-version informed consent after clarifying the relevant information on the study objectives, benefits, medical procedures, confidentiality agreement on personal information, and agreement on publication of related data research.

### Lifestyle risk factors

Information, such as age, gender, or physical activity, was obtained from a standard questionnaire during the interview. We also asked the participants whether they were currently smoking or drinking. Physical activity level was considered to combine occupational workload and leisure-time exercise and was then reclassified into three levels: low, moderate, and high level. The salt intake was classified into three categories, low, medium, or high salt intake, defined as ≤6, 6–10, or >10 g/day, respectively. We used tea consumption to represent the caffeine intake of the population and divided the population into three subgroups: no tea consumption subgroup, rarely subgroup (one to two cups/day) or often subgroup (≥3 cups/day). All participants were asked whether they had suffered from CVD, stroke, or kidney diseases.

### Variable measurement

All participants were told to fast for at least 12 h in advance, and blood samples were collected the next morning. The blood samples were added to vacutainer tubes containing an anticoagulant, and plasma was obtained by centrifugation. The final purpose of blood samples was to gather data on blood biochemistry and perform blood routine examinations. Fasting blood glucose (FPG), triglyceride (TG), plasma total cholesterol (TC), low-density lipoprotein cholesterol (LDL-C), high-density lipoprotein cholesterol (HDL-C), and glycated hemoglobin (HbA1C) were obtained by enzymatic analysis on an Olympus AU640 automated analyzer (Olympus, Kobe, Japan). All laboratory equipment was calibrated, and samples were repeated using the blind method. The measurement of height and weight needed participants to keep a standing posture, wear lightweight clothes, and be without shoes.

The waist circumference (WC) was measured at the umbilicus level at the end of a normal expiration. The measurement results were accurate to 0.1 kg and 0.1 cm, respectively. The measurement of blood pressure was performed according to the American Heart Association protocol. Subjects were told to rest in a quiet room for at least 10 min, and an automatic electronic sphygmomanometer (HEM-741C; Omron, Tokyo, Japan) was used to measure blood pressure three times. The measurements were taken on the naked upper arm in a seated position with a 5-min interval between measurements. The average of three blood pressure measurements was selected and used for all subsequent analyses.

### Definition

According to the 7th Joint National Committee guideline, we defined hypertension as blood pressure of ≥140/90 mmHg (systolic/diastolic blood pressure [SBP/DBP]) or being under medication treatment for hypertension in the last 2 weeks (25). DM was defined as FPG of ≥7.0 mmol/l or a previous diagnosis of DM (26). Hyperuricemia was diagnosed as serum uric acid (SUA) concentration of ≥420 μmol/l for men and ≥360 μmol/l for women (27). According to the International Diabetes Federation (IDF) definition (28), MetS was defined by central obesity (waist circumference of ≥90 cm for men and ≥80 cm for women) plus any two of the following factors: 1) TG of ≥1.7 mmol/l; 2) HDL-C level of <1.03 mmol/l in men or <1.29 mmol/l in women, or specific treatment for this lipid abnormality; 3) SBP of ≥130 or DBP of ≥85 mmHg or treatment of previously diagnosed hypertension; 4) FPG of ≥5.6 mmol/l or previously diagnosed T2DM. Dyslipidemia was defined by satisfying any of the following diseases (29): (1) hypercholesterolemia: plasma total cholesterol (TCH) of ≥5.2 mmol/l; (2) hypertriglyceridemia: TG of ≥1.7 mmol/l; (3) high LDL-C: LDL-C of ≥3.4 mmol/l; (4) low HDL-C: HDL-C of <1.03 mmol/l for men or <1.29 mmol/l for women. MetD was defined by satisfying any of MetS, dyslipidemia, and central obesity criteria. As for the estimated glomerular filtration rate (eGFR), we chose formulas including creatinine level suggested by the Chronic Kidney Disease Epidemiology Collaboration equations (CKD-EPI) (30). The body mass index (BMI) was calculated as weight (kg)/height (m^2^). The 10-year CVD risk was determined by the overall score of the Framingham risk score (FRS), which included gender, age, HDL-C, TCH, SBP, and smoking (31). Furthermore, the inflammatory indicators were calculated by the following: 1) MHR = monocyte count/HDL-C (15); 2) NLR = neutrophil count/lymphocyte count (15); 3) SII = platelet count × neutrophil count/lymphocyte count (16); 4) SIRI = neutrophil count × monocyte count/lymphocyte count (17).

We also converted four inflammatory indicators into three levels by tertiles, and the lowest level was set as a reference. The detailed intervals of these indicators were as follows: 1) MHR, T1: ≤0.27; T2: 0.28–0.42; T3: ≥0.43; 2) NLR, T1: ≤1.47; T2: 1.48–2.08; T3: ≥2.09; 3) SII, T1: ≤282.63; T2: 282.64–427.34; T3: ≥427.38; 4) SIRI, T1: ≤0.61; T2: 0.62–1.03; T3: ≥1.04.

### Statistical analysis

Overall, the data were normally distributed; thus, we described them by mean ± standard deviation (M ± SD) or frequency and percentage for continuous and categorical variables, respectively. The differences between continuous variables were compared by one-way analysis of variance (ANOVA) and χ^2^-test analysis for categorical variables. We conducted a multivariable logistic regression model to calculate odd ratios (ORs) and 95% confidence interval (CI) to be able to assess the association between inflammatory indicators and various diseases. Statistical analyses were performed by SPSS software version 22.0 (IBM Corp., Armonk, NY, USA). A P value of <0.05 under the two-tailed condition was considered statistically significant.

## Results

### The study subjects had a high prevalence of chronic diseases and metabolic diseases

**Tables 1**, **2** summarize the characteristic of participants in the present study. The subjects involved in the present study were predominantly middle-aged and elderly population with an average age of 53.7 years (54.1 years for men and 53.39 years for women). Our study subjects were exposed to multiple CVD risk factors, leading to prevalent chronic and metabolic diseases; for example, 15.6% of the study population had CVD (19.4% for men and 11.0% for women), and 9.3% of participants suffered from stroke (9.8% for men and 8.9% for women). On the manifestation of chronic diseases, the subjects also had an obviously elevated BP level (138.54/82.24 mmHg averagely, 140.9/84.16 mmHg for men, and 136.6/80.65 mmHg for women) and FPG level (5.96 mmol/l averagely, 6.02 mmol/l for men, and 5.91 mmol/l for women), which consequentially caused the high prevalence of hypertension and DM in both genders (hypertension, 50.6% for men and 44.2% for women; DM, 10.81% for men and 11.8% for women). Most importantly, we observed that participants had prevalent metabolic disorders, where around 80.4% of participants suffered from it (71.4% for men and 87.8% for women).

**Table 1:** Risk factor characteristics of the baseline population.

	Total N = 7,420	Male N = 3,359	Female N = 4,061	pvalue
	N (%)	N (%)	N (%)	
MetD	5,963 (80.4)	2,397 (71.4)	3,566 (87.8)	<0.001
MetS	2,317 (31.2)	680 (20.2)	1,637 (40.3)	<0.001
Dyslipidemia	5,613 (75.6)	2,261 (67.3)	3,352 (82.5)	<0.001
Hypercholesterolemia	3,781 (51.0)	1,595 (47.5)	2,186 (53.8)	<0.001
Hypertriglyceridemia	2,465 (33.2)	1,103 (32.8)	1,362 (33.5)	0.523
High LDL-C	1,827 (24.6)	729 (21.7)	1,098 (27.0)	<0.001
Low HDL-C	2,512 (33.9)	600 (17.9)	1,912 (47.1)	<0.001
Central obesity	3,040 (41.0)	842 (25.1)	2,198 (54.1)	<0.001
History of CVD	1,158 (15.6)	370 (11.0)	788 (19.4)	<0.001
History of stroke	692 (9.3)	330 (9.8)	362 (8.9)	0.180
History of nephrosis	150 (2.0)	55 (1.6)	95 (2.3)	0.032
Hypertension	3,494 (47.1)	1,699 (50.6)	1,795 (44.2)	<0.001
DM	842 (11.3)	363 (10.8)	479 (11.8)	0.182
Hyperuricemia	929 (12.5)	574 (17.1)	355 (8.7)	<0.001
Current smoking	2,560 (34.5)	1,938 (57.7)	622 (15.3)	<0.001
Current drinking	1,599 (21.5)	1,507 (44.9)	92 (2.3)	<0.001
Physical activity				<0.001
Low	2,722 (36.9)	943 (28.2)	1,779 (44.2)	
Medium	1,402 (19.0)	635 (19.0)	767 (19.0)	
High	3,247 (44.1)	1,765 (52.8)	1,482 (36.8)	
Salt intake				0.003
Low (<6 g/day)	86 (1.2)	27 (0.8)	27 (1.2)	
Medium (≥6 and <10 g/day)	1,109 (15.0)	470 (14.0)	639 (15.8)	
High (≥10 g/day)	6,203 (83.8)	2,851 (85.2)	3,352 (82.8)	
Tea consumption				<0.001
No	5,456 (73.5)	2,015 (60.0)	3,441 (84.7)	
Rarely (1–2 cups/day)	1,862 (25.1)	1,258 (37.5)	604 (14.9)	
Often (≥3 cups/day)	102 (1.4)	86 (2.6)	16 (3.9)	

Data are presented as N (%). Statistical significance was defined at p < 0.05 under two-tailed conditions. CVD, cardiovascular disease; DM, diabetes mellitus; HDL-C, high-density lipoprotein cholesterol; LDL-C. low-density lipoprotein cholesterol; MetD, metabolic disorders; MetS, metabolic syndrome.

**Table 2:** The anthropometric and biochemical parameters of the baseline population.

	Total N = 7,420	Male N = 3,359	Female N = 4,061	pvalue
	M ± SD	M ± SD	M ± SD	
Age (years)	53.71 ± 8.68	54.10 ± 8.74	53.39 ± 8.62	0.42
BMI (kg/m^2^)	24.58 ± 3.55	24.47 ± 3.43	24.67 ± 3.64	0.0012
WC (cm)	81.79 ± 9.61	82.93 ± 9.62	80.84 ± 9.49	<0.001
SBP (mmHg)	138.54 ± 21.62	140.90 ± 21.15	136.60 ± 21.81	<0.001
DBP (mmHg)	82.24 ± 11.63	84.16 ± 11.57	80.65 ± 11.45	<0.001
WBC (×10^9^/L)	6.18 ± 2.08	6.43 ± 2.26	5.98 ± 1.90	<0.001
Monocyte (×10^9^/L)	0.48 ± 0.28	0.50 ± 0.28	0.46 ± 0.28	<0.001
Neutrophil (×10^9^/L)	3.74 ± 2.85	3.92 ± 2.84	3.58 ± 2.85	<0.001
Lymphocyte (×10^9^/L)	2.66 ± 2.22	2.77 ± 2.25	2.57 ± 2.19	0.333
Platelet (×10^9^/L)	206.37 ± 60.87	195.65 ± 58.36	215.23 ± 61.48	<0.001
BUN (mmol/L)	5.48 ± 2.19	5.81 ± 2.14	5.22 ± 2.19	<0.001
Scr (μmol/L)	75.45 ± 22.05	82.80 ± 20.31	69.37 ± 21.58	<0.001
eGFR (mL/min/1.73 m^2^)	89.73 ± 14.59	91.80 ± 14.58	88.01 ± 14.38	<0.001
SUA (μmol/L)	299.15 ± 84.94	342.60 ± 84.33	263.20 ± 77.69	<0.001
FPG (mmol/L)	5.96 ± 1.55	6.02 ± 1.62	5.91 ± 1.50	0.002
TCH (mmol/L)	5.33 ± 1.10	5.25 ± 1.07	5.40 ± 1.13	<0.001
TG (mmol/L)	1.69 ± 1.49	1.70 ± 1.59	1.69 ± 1.41	0.672
HDL-C (mmol/L)	1.34 ± 0.32	1.33 ± 0.34	1.35 ± 0.30	0.019
LDL-C (mmol/L)	2.90 ± 0.80	2.85 ± 0.78	2.94 ± 0.81	<0.001
MHR	0.38 ± 0.25	0.36 ± 0.25	0.40 ± 0.24	<0.001
NLR	1.90 ± 0.99	1.83 ± 0.85	1.99 ± 1.13	<0.001
SII (*10^9^/L)	390.19 ± 227.63	385.67 ± 233.48	393.92 ± 222.63	0.120
SIRI (*10^9^/L)	0.93 ± 0.89	0.96 ± 0.87	1.21 ± 0.80	<0.001

Data are presented as M ± SD. Statistical significance was defined at p < 0.05 under two-tailed conditions. BMI, body mass index; BUN, blood urea nitrogen; DBP, diastolic blood pressure; eGFR, estimated glomerular filtration rate; FPG, fasting plasma glucose; HDL-C, high-density lipoprotein cholesterol; LDL-C. low-density lipoprotein cholesterol; M, mean; MHR, monocyte-to-high-density lipoprotein ratio; NLR, neutrophil-to-lymphocyte ratio; SBP, systolic blood pressure; Scr, serum creatinine; SD, standard deviation; SII, systematic immune-inflammation index; SIRI, systemic inflammation response index; SUA, serum uric acid; TCH, plasma total cholesterol; TG, triglyceride; WBC, white blood cells; WC, waist circumference.

Among the components of metabolic disorders, the prevalence of MetS was 31.2%, that of central obesity was 33.2%, and that of dyslipidemia was 75.6%. After the detailed classification of dyslipidemia, we found that 51% of participants had hypercholesterolemia, 33.2% had hypertriglyceridemia, and the prevalence of high LDL and low HDL was 24.6% and 33.9%, respectively. We also observed that female participants seemed more likely to suffer from metabolic disorders or various abnormal metabolic statuses than men. In terms of lifestyle, smoking and drinking accounted for 34.5% and 21.5% of participants, respectively, and were both more prevalent among the male population (57.7% vs. 15.3% for current smoking and 44.9% vs.2.3% for current drinking). Furthermore, the male population generally had a higher intensity of physical activity (52.8%), and female participants mostly had a low physical activity level (44.2%). In terms of diet, we noticed that male participants had a slight higher salt intake (≥10 g/day: 85.2% in men vs. 82.8% in women) and female participants presented a regular tea consumption (2.6% in men vs. 3.9% in women). We enrolled four inflammation parameters to systematically evaluate the inflammation status in the present study and found that male subjects had a lower inflammation degree than women (MHR, 0.36 vs. 0.40; NLR, 1.83 vs. 1.99; SIRI, 0.96 vs. 1.21), showing a similar difference in gender with the prevalence of various metabolic abnormalities.

### The subjects with metabolic diseases had higher levels of inflammation status

We divided the subjects into various subgroups according to different metabolic diseases and compared the inflammation status difference between the normal subgroup and the corresponding disease subgroup to clarify whether inflammation had a potential association with metabolic status (**Table 3**). Overall, we observed that each indicator showed a significant increase in MetD patients. Additionally, in the components of MetD, we found that MetS patients had a higher level of all four inflammation parameters than non-MetS patients, but we only found that MHR, SII, and SIRI were significantly increased in dyslipidemia patients, and only MHR and SIRI were significantly elevated in central obesity patients. After further classification of dyslipidemia, we found that every four indicators in both hypercholesterolemia and hypertriglyceridemia patients showed a significant increment. The subjects with high LDL-C showed an overall increased inflammation marker levels except for MHR. We also found that MHR, SII, and SIRI were significantly increased in the subjects with low HDL-C. The above results suggested that abnormal metabolic status was usually accompanied by elevated inflammation parameters and showed higher inflammation levels than normal.

**Table 3:** The different levels of inflammation parameters divided by metabolic disorders or their components.

	MHR	NLR	SII	SIRI
	M ± SD	M ± SD	M ± SD	M ± SD
MetD				
Yes	0.49 ± 0.25	1.96 ± 1.00	392.60 ± 225.82	1.24 ± 0.83
No	0.33 ± 0.22	1.89 ± 0.98	370.30 ± 234.70	0.82 ± 0.62
p	<0.001	0.017	<0.001	<0.001
MetS				
Yes	0.44 ± 0.28	1.92 ± 1.07	391.40 ± 238.51	0.97 ± 1.09
No	0.36 ± 0.23	1.27 ± 0.80	379.64 ± 201.63	0.92 ± 0.79
p	<0.001	0.027	0.042	0.018
Dyslipidemia				
Yes	0.40 ± 0.25	1.89 ± 0.97	394.10 ± 226.95	0.94 ± 0.83
No	0.34 ± 0.23	1.94 ± 1.07	378.03 ± 229.36	0.82 ± -.61
p	<0.001	0.051	0.009	<0.001
Central obesity				
Yes	0.42 ± 0.28	1.87 ± 1.06	389.20 ± 210.27	0.95 ± 1.06
No	0.36 ± 0.22	1.93 ± 0.86	390.87 ± 238.96	0.92 ± 0.75
p	<0.001	0.757	0.163	0.011
Hypercholesterolemia				
Yes	0.56 ± 0.24	1.96 ± 0.94	391.70 ± 230.29	1.39 ± 1.00
No	0.40 ± 0.20	1.55 ± 1.04	368.61 ± 224.85	0.94 ± 0.76
p	<0.001	<0.001	<0.001	<0.001
Hypertriglyceridemia				
Yes	0.45 ± 0.28	1.91 ± 1.06	394.32 ± 207.53	1.22 ± 1.00
No	0.35 ± 0.23	1.88 ± 0.85	378.13 ± 236.98	0.90 ± 0.76
p	<0.001	0.021	<0.001	<0.001
High LDL-C				
Yes	0.37 ± 0.26	1.92 ± 1.02	401.50 ± 216.32	1.39 ± 1.13
No	0.38 ± 0.24	1.54 ± 0.89	386.49 ± 231.10	0.94 ± 0.80
p	0.089	0.01	<0.001	<0.001
Low HDL-C				
Yes	0.47 ± 0.29	1.90 ± 0.84	400.04 ± 216.62	1.10 ± 0.95
No	0.34 ± 0.21	1.90 ± 1.06	385.15 ± 232.92	0.93 ± 0.76
p	<0.001	0.429	<0.001	<0.001

Data are presented as M ± SD. Statistical significance was defined at p < 0.05 under two-tailed conditions. HDL-C, high-density lipoprotein cholesterol; LDL-C. low-density lipoprotein cholesterol; M, mean; MetD, metabolic disorders; MetS, metabolic syndrome; MHR, monocyte-to-high-density lipoprotein ratio; NLR, neutrophil-to-lymphocyte ratio; SD, standard deviation; SII, systematic immune-inflammation index; SIRI, systemic inflammation response index.

### The prevalence of metabolic diseases was elevated with gradually aggravating inflammation status

**Figure 2:**
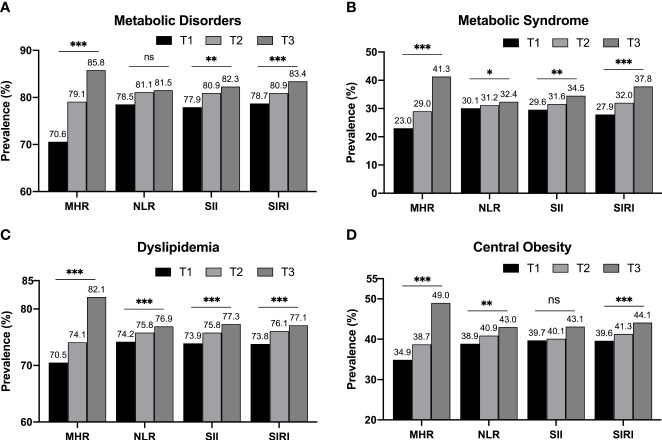
The prevalence of metabolic diseases in different inflammatory indicators grouped by tertiles. Regardless of the total MetD **(A)** or its detailed components, such as MetS **(B)**, dyslipidemia **(C)**, and central obesity **(D)**, they all showed a gradually increasing tendency with each tertile increment of various four inflammatory indicators. Statistical significance was defined under two-tailed condition. *p < 0.05; **p < 0.01; ***p < 0.001; ns, no significant.

We divided each inflammatory indicator into three levels by tertiles and compared the prevalence of metabolic diseases among different tertiles to clarify the changes in the risk of metabolic diseases under different inflammatory conditions (**Figure 2**). We observed that the general trend of alterations in MetD prevalence and its components were similar and consistent, showing a gradually elevated level with increased tertiles of four inflammatory parameters. Among these indicators, we observed that the tertile increment of MHR and SIRI caused a stable and consistent elevation in the prevalence of various metabolic diseases. We also noticed that the magnitudes of increment in MHR were larger and more moderate in SIRI. The prevalence of MetD in the T3 subgroup of NLR had increased only by 0.4% based on the T2 subgroup, and there was only a 0.4% incremental prevalence of central obesity between the T1 and T2 subgroups of SII. Only the T3 subgroup had a significantly increased prevalence (by 3.0%). The abovementioned imbalanced alterations led to a statistically insignificant trend in their overall alterations. These alteration tendencies indicated that the subjects with a higher level of inflammatory parameters seemed more likely to have metabolic diseases, suggesting that there might be an association between inflammation and metabolic diseases.

### High level of inflammatory parameters was associated with a higher risk of having metabolic diseases

To reveal the association between inflammation and metabolic diseases, we conducted multivariable logistic regression and adjusted different co-variables to guarantee the accuracy and reliability of the association (**Table 4**). For MetD and MetS, we adjusted the ORs of different inflammatory parameters by model A, which included age, gender, a history of CVD, WBC counting, SUA concentration, eGFR, physical activity, salt intake, tea consumption, and a history of current smoking and drinking. For dyslipidemia, we added hypertension and DM based on model A and defined it as model B. For central obesity, we set up model C, which supplemented TCH, TG, LDL-C, and HDL-C into model B. Overall, we observed a significant association between inflammation and the risk of having metabolic diseases, which included MetD and various components of it, and the risk trended to elevate with increasing levels of inflammation. Compared with the lowest inflammatory indicator subgroup (T1 subgroups of all indicators), which was defined as a reference, the T3 subgroups of MHR had a 2.653-fold higher risk of MetD (the highest risk) (OR, 2.653; 95% CI, 2.142–3.286) and a 1.547-fold higher risk of having dyslipidemia (the highest risk) (OR, 1.547; 95% CI, 1.287–1.859) than reference.

**Table 4:** Inflammation states are associated with the risk of metabolic disorders and their components.

	Inflammation indicators tertiles			Continuous
	T1	T2	T3	per SD increment
	Reference	OR (95% CI)	OR (95% CI)	OR (95% CI)
MetD ^a^				
MHR	Reference	1.385 (1.176-1.632) ^#^	2.653 (2.142-3.286) ^#^	1.443 (1.240-1.679) ^#^
NLR	Reference	1.127 (0.926-1.372)	1.031 (0.783-1.358)	1.052 (0.811-1.366)
SII	Reference	1.105 (0.948-1.287)	1.299 (1.126-1.500) *	1.116 (1.049-1.188) *
SIRI	Reference	1.361 (1.174-1.577) ^#^	2.092 (1.622-2.699) ^#^	1.378 (1.047-1.815) *
MetS ^a^				
MHR	Reference	1.424 (1.071-1.895) *	2.091 (1.620-2.698) ^#^	1.376 (1.310-1.444) ^#^
NLR	Reference	1.011 (0.837-1.220)	1.174 (0.954-1.445)	1.093 (0.849-1.407)
SII	Reference	1.267 (1.019,1.576) *	1.588 (1.324-1.904) ^#^	1.235 (1.112-1.372) ^#^
SIRI	Reference	1.592 (1.376-1.843) ^#^	3.441 (2.917-4.058) ^#^	1.659 (1.413-1.947) ^#^
Dyslipidemia ^b^				
MHR	Reference	1.131 (1.038-1.232) *	1.547 (1.287-1.859) ^#^	1.472 (1.238-1.752) *
NLR	Reference	1.031 (0.830-1.281)	0.999 (0.992-1.007)	0.986 (0.967-1.005)
SII	Reference	1.221 (1.054-1.416) *	1.367 (1.176-1.591) ^#^	1.112 (1.030-1.200) *
SIRI	Reference	1.237 (1.081-1.418) *	1.417 (1.218-1.649) ^#^	1.197 (1.176-1.218) ^#^
Central obesity ^c^				
MHR	Reference	1.085 (0.855-1.377)	1.515 (1.389-1.652) ^#^	1.418 (1.342-1.499) ^#^
NLR	Reference	1.093 (0.849-1.407)	1.194 (0.957-1.490)	1.195 (0.950-1.504)
SII	Reference	1.120 (1.052-1.192) ^#^	1.299 (1.190-1.418) ^#^	1.216 (1.010-1.160) *
SIRI	Reference	1.445 (1.369-1.525) ^#^	2.080 (1.613-2.683) ^#^	1.454 (1.211-1.730) ^#^

Statistical significance was defined at the following: *p < 0.05 under two-tailed conditions; ^#^p < 0.001 under two-tailed conditions. a, the regression model that included age, gender, history of CVD, WBC counting, SUA concentration, eGFR, physical activity, salt intake, tea consumption, and current smoking and drinking. b, the regression model that included age, gender, history of CVD, WBC counting, SUA concentration, eGFR, physical activity, salt intake, tea consumption, current smoking and drinking, hypertension, and DM. c, the regression model that included age, gender, history of CVD, WBC counting, SUA concentration, eGFR, TCH level, TG level, LDL-C level, HDL-C level, physical activity, salt intake, tea consumption, current smoking and drinking, hypertension, and DM. The detailed intervals of these indicators were as the following: (1) MHR, T1: ≤0.27; T2: 0.28–0.42; T3: ≥0.43; (2) NLR, T1: ≤1.47; T2: 1.48–2.08; T3: ≥2.09; (3) SII, T1: ≤282.63; T2: 282.64–427.34; T3: ≥427.38; (4) SIRI, T1: ≤0.61; T2: 0.62–1.03; T3: ≥1.04. CI, confidence interval; HDL-C, high-density lipoprotein cholesterol; LDL-C. low-density lipoprotein cholesterol; M, mean; MetD, metabolic disorders; MetS, metabolic syndrome; MHR, monocyte-to-high-density lipoprotein ratio; NLR, neutrophil-to-lymphocyte ratio; OR, odds ratio; SD, standard deviation; SII, systematic immune-inflammation index; SIRI, systemic inflammation response index; T, tertile.

The highest risk of MetS and central obesity was observed in the T3 subgroup of SIRI, with a risk of 3.441-fold (OR, 3.441; 95% CI, 2.917–4.058) for MetS and 2.080-fold (OR, 2.080; 95% CI, 1.613–2.683) for central obesity. By comparing the various indicators of the present study, we found that MHR and SIRI had a stable ability to evaluate the risk of metabolic diseases, showing a gradually elevated risk of having MetD or its components in every increased level of these two indicators, leading to a linear relationship between the levels of these two indicators and the risk of metabolic diseases. Each SD increment of MHR caused a 44.3% increase in risk for MetD, a 37.6% additional risk for MetS, a 47.2% additional risk for dyslipidemia, and a 41.8% additional risk for central obesity. Furthermore, each SD increment of SIRI caused a 37.8% extra risk for MetD, a 65.9% extra risk for MetS, a 19.7% extra risk for dyslipidemia, and a 45.4% extra risk for central obesity.

SII also showed good ability in risk evaluation of various metabolic diseases, such as MetS, dyslipidemia, and central obesity, and the tendencies of elevated risk also showed a linear association with per SD increment of SII. However, we did not observe the association between the T2 level of SII and the risk of MetD, leading to the non-linear risk association between SII and the risk of MetD. Lastly, NLR did not display an evaluation of the risk of various metabolic diseases at almost all levels.

### High levels of inflammatory status predicted a higher risk of CVD

We calculated the FRS for each participant in these tertile subgroups and determined the 10-year CVD risk by their total score to further explain the applicability of these inflammatory indicators in predicting adverse outcomes (**Table 5**). We observed a progressive increment in CVD development risk with increasing inflammatory levels, and the T3 subgroups of all inflammatory indicators showed the highest risk of 10-year CVD, which was 35%–40% in the over 10%/11% (men/women) risk subgroup, 10%–15% in the over 20%/22% (men/women) risk subgroup and approximately 3.0% in the over 30% risk subgroup. In addition, by comparing the alteration trend and per-level increment, we found that the inflammation level increase from T2 to T3 would bring a greater increment risk of CVD, especially in the over 20/22% and over 30% subgroups of FRS. These results demonstrated that the high inflammation levels indeed tended to indicate a higher risk of CVD or metabolic diseases or suggested that the subject was exposed to a high-risk status of diseases.

**Table 5:** Ten-year CVD risk reflected by FRS among different levels of multiple inflammation indicators.

	Ten-year CVD risk					
	Men >10%/women >11%		Men >20%/women >22%		Both >30%	
	N	%	N	%	N	%
MHR						
T1	632	25.7	156	6.4	22	0.9
T2	757	31.1	202	8.3	31	1.3
T3	1017	40.1	399	15.7	90	3.6
p for trend	<0.001		<0.001		<0.001	
NLR						
T1	682	27.9	198	8.1	32	1.3
T2	820	32.7	246	9.8	44	1.8
T3	904	36.6	313	12.7	67	2.7
p for trend	<0.001		<0.001		<0.001	
SII						
T1	782	31.6	232	9.4	36	1.5
T2	784	31.7	254	10.3	55	2.2
T3	840	34.0	321	13.0	79	3.2
p for trend	0.007		0.025		<0.001	
SIRI						
T1	597	24.2	156	6.3	21	0.9
T2	848	33.0	242	9.4	48	1.9
T3	961	40.4	359	15.1	74	3.1
p for trend	<0.001		<0.001		<0.001	

The detailed intervals of these indicators were as the following: (1) MHR, T1: ≤0.27; T2: 0.28–0.42; T3: ≥0.43; (2) NLR, T1: ≤1.47; T2: 1.48–2.08; T3: ≥2.09; (3) SII, T1: ≤282.63; T2: 282.64–427.34; T3: ≥427.38; (4) SIRI, T1: ≤0.61; T2: 0.62–1.03; T3: ≥1.04. CVD, cardiovascular disease; FRS, Framingham risk score; MHR, monocyte-to-high-density lipoprotein ratio; NLR, neutrophil-to-lymphocyte ratio; SII, systematic immune-inflammation index; SIRI, systemic inflammation response index; T, tertile.

Statistical significance was defined under two-tailed conditions.

### Chronic inflammatory status was accompanied by a higher risk of 10-year CVD

Previous results have already confirmed that high levels of inflammatory parameters tended to bring a higher CVD risk. Based on this hypothesis, we further conducted a logistic regression model of CVD risk, which included gender, current drinking, physical activity, salt intake, tea consumption, DM, hyperuricemia, WBC counting, BUN, Scr, TG, LDL-C, and inflammatory indicators, to confirm whether the high level of inflammation status was indeed associated with elevated CVD risk (**Table 6**). We observed that the subjects having a higher inflammation level were likely to have a higher risk of CVD, especially for the 10-year CVD risk in the over 10%/11% risk subgroup, in which the highest tertiles of all four indicators showed a significant association with it. Among these indicators, MHR and SIRI exhibited a stable correlation with CVD risk, and NLR and SII did not show a significant association with 10-year CVD risk and merely showed a weak association in some highest tertiles.

**Table 6:** Chronic inflammation status was associated with 10-year CVD risk.

	Adjusted 10-year CVD risk					
	Men >10%/women >11%		Men >20%/women >22%		Both >30%	
	ORs (95% CI)	p value	ORs (95% CI)	p value	ORs (95% CI)	p value
MHR						
T1	–	–	–	–	–	–
T2	1.332 (1.090-1.628)	0.005	1.891 (1.490-2.400)	0.039	1.832 (0.984-3.413)	0.056
T3	1.937 (1.592-2.358)	<0.001	2.696 (2.119-3.431)	<0.001	4.607 (2.648-8.017)	<0.001
Per SD increment	1.293 (1.194–1.400)	<0.001	1.423 (1.305-1.551)	<0.001	1.468 (1.310-1.647)	<0.001
NLR						
T1	–	–	–	–	–	–
T2	1.119 (0.983-1.440)	0.075	1.098 (0.868-1.389)	0.435	1.218 (0.738-2.012)	0.440
T3	1.290 (1.063-1.565)	0.010	1.349 (1.074-1.695)	0.010	1.740 (0.827–2.779)	0.021
Per SD increment	1.022 (0.953–1.095)	0.544	1.074 (1.000-1.153)	0.051	1.094 (0.979-1.222)	0.113
SII						
T1	–	–	–	–	–	–
T2	1.130 (0.935-1.365)	0.207	1.123 (0.896-1.408)	0.314	1.528 (0.963-2.426)	0.072
T3	1.265 (1.046-1.531)	0.016	1.166 (0.933-1.456)	0.177	1.292 (1.206-1.383)	<0.001
Per SD increment	1.061 (0.983–1.146)	0.130	1.041 (0.858-1.263)	0.681	1.134 (0.980-1.311)	0.092
SIRI						
T1	–	–	–	–	–	–
T2	1.372 (1.129–1.667)	0.001	1.315 (1.014-1.704)	<0.001	2.071 (1.1623-3.692)	0.014
T3	1.568 (1.290–1.907)	<0.001	2.488 (2.212-2.797)	<0.001	3.397 (1.958-5.894)	<0.001
Per SD increment	1.220 (1.205–1.236)	<0.001	1.326 (1.255-1.401)	<0.001	1.557 (1.228-1.974)	<0.001

Logistic regression model of CVD risk: gender, current drinking, physical activity, salt intake, tea consumption, DM, hyperuricemia, WBC counting, BUN, Scr, TG, LDL-C, and inflammatory indicators, respectively. CI, confidence interval; CVD, cardiovascular disease; HDL-C, high-density lipoprotein cholesterol; LDL-C, low-density lipoprotein cholesterol; M, mean; MetD, metabolic disorders; MetS, metabolic syndrome; MHR, monocyte-to-high-density lipoprotein ratio; NLR, neutrophil-to-lymphocyte ratio; OR, odds ratio; SD, standard deviation; SII, systematic immune-inflammation index; SIRI, systemic inflammation response index; T, tertile.

Statistical significance was defined under two-tailed conditions.

Compared with the lowest tertile, the subjects in the highest tertile of MHR had a 1.937-fold higher risk for 10-year CVD risk in the over 10%/11% risk subgroup, a 2.696-fold higher risk for 10-year CVD risk in the over 20%/22% risk subgroup, and a 4.607-fold higher risk for 10-year CVD risk in the over 30% risk subgroup. As for SIRI, the subjects in the highest tertile of SIRI had a 1.568-fold higher risk for 10-year CVD risk in the over 10%/11% risk subgroup, a 2.488-fold higher risk for 10-year CVD risk in the over 20%/22% risk subgroup, and a 3.397-fold higher risk for 10-year CVD risk in the over 30% risk subgroup compared to the lowest tertile. Furthermore, each SD increment of MHR caused a 29.3% additional risk for 10-year CVD risk in the over 10%/11% risk subgroup, a 42.3% extra risk for 10-year CVD risk in the over 20%/22% risk subgroup, and a 46.8% additional risk for 10-year CVD risk in the over 30% risk subgroup. In addition, SIRI displayed a more aggravated and stable elevated tendency in 10-year CVD risk, each SD increment of SIRI could bring a 22.0% additional risk in the over 10%/11% subgroup, 32.6% in the over 20%/22% subgroup, and 55.7% in the over 30% subgroup. These results suggested that a higher inflammatory status was associated with a higher CVD risk, confirming our hypothesis that long-term chronic inflammation seemed likely to cause CVD or elevate the CVD risk in the future.

## Discussion

Most current studies focused on inflammation and metabolism have been conducted at the cellular level and indicated the regulation patterns and interaction effects between various cellular inflammatory factors and molecular metabolic behaviors. Our results, for the first time, revealed the epidemiological association between inflammation levels and the risk of metabolic diseases in a large-scale rural population in China. Our results filled a gap in the study of inflammation and metabolism at the individual and population levels, demonstrating that long-term chronic inflammatory states also affect metabolic status at the individual level, and higher levels of inflammation in the population were significantly associated with an elevated risk of various metabolic diseases, such as MetD and its components. We also compared and screened various inflammatory indicators and found that MHR and SIRI demonstrated a significant and stable effect in evaluating the risk of metabolic diseases in which a higher-level inflammation status was usually accompanied by a higher risk of having MetD and its components. Lastly, based on the metabolic diseases, we further clarified the association between inflammation and risk of CVD and found that a higher inflammatory status was indeed associated with an elevated 10-year CVD risk, suggesting that long-term chronic inflammation seemed likely to cause CVD or elevate the CVD risk in future. Thus, we believe that high inflammation levels tended to indicate a higher risk of CVD or metabolic diseases or suggested that the subject was exposed to a high-risk status of diseases.

In the present study, we used four parameters to reflect inflammatory status. MHR and NLR were traditional indicators to evaluate the inflammatory status. SII and SIRI were novel indicators that assessed the balance between systemic inflammation and immune response in the body and had a better effect on reflecting the inflammatory state (17). Previous studies have confirmed that these indicators were more comprehensive and effective in evaluating inflammation levels and correlated with the prognosis of multiple chronic diseases and adverse CVD events (18–20, 32). Among four indicators, we observed that MHR and SIRI expressed a significant association with metabolic diseases, both overall MetD and detailed MetS, dyslipidemia, and central obesity. We also found that the risk association between these two inflammatory indicators and various metabolic diseases was in a linear manner, in which the risk would gradually elevate with the increment of indices. We observed that SII failed to have a significant association with MetD under low-grade inflammatory status (T2 subgroup). Regarding other metabolic disease components, compared with MHR or SIRI, SII had a weak but significant association with the risk of having MetS, dyslipidemia, and central obesity. Consistent with our conclusion, previous studies have observed that MRH might have a potential linkage with metabolic status. Some studies have indicated that the MHR was associated with BMI and WC levels among MetS patients and observed that patients usually had a higher MHR level, although MetS patients showed low-grade inflammatory status (33). Moreover, the elevated MHR level has been correlated with dyslipidemia among patients with chronic obstructive pulmonary disease (34). SII and SIRI have recently become novel parameters, with which a sufficient number of studies in the metabolic yield have not been yet conducted, but a study focused on the rural population believed that SIRI was correlated with hyperuricemia and SIRI could optimize the risk stratification of hyperuricemia (35), which is also a type of metabolic diseases, indirectly supporting our conclusion. Additionally, a study has revealed that SII had a significant positive linear correlation with increased BMI, which could predict the risk for obesity (36). Therefore, we believe that SIRI had a robust association with metabolic diseases and could assess the risk of MetD and its components, especially MetS.

We observed that NLR almost did not show any association with the risk of metabolic diseases, regardless of the variable (category or continuous). However, we found that NLR demonstrated a significant correlation with MetS or related metabolic outcomes in some studies of specific populations, such as obesity population, bipolar disorder patients, and hyperglycemia patients during pregnancy (37–41). Additionally, patients with multiple metabolic diseases tend to have higher levels of NLR (42). We compared the population characteristics of these studies and further compared the differences between the four different inflammatory indicators. Thus, we believe that there were two possible reasons for these various conclusions. First, we found that NLR was commonly used in multiple-malignancy research to reflect the correlation between inflammation status and the risk of adverse prognosis, in which the microenvironment of patients was already in an overactive or extremely imbalanced inflammatory status (21, 22, 43). We also noticed that the subjects of these previous studies on NLR and metabolic studies all had different levels of metabolic abnormalities, suggesting that NLR was more appropriate for the evaluation of inflammatory status in populations with high inflammation levels. Hence, we believe that NLR seemed to be more suitable for evaluating the risk of metabolic or other adverse events based on preexisting severe diseases, such as MetS, obesity, and DM. We noticed that the calculation of NLR only involved neutrophil and lymphocyte counts, which could only simply respond to the inflammatory state, but MHR and SIRI additionally involved monocyte counts, suggesting that neutrophil counting alone did not provide a good evaluation of metabolic diseases and the immune state represented by monocytes seemed to play an important role in metabolic dysfunction. Thus, we believe that the immune-inflammatory system might be the real participant in inflammatory factor-mediated metabolic alteration. Moreover, we found that although SII did not directly involve monocyte counts, the additional factor of platelet counting could provide a certain description of the immune status to this indicator, allowing us to observe a correlation between SII and multiple metabolic diseases. Due to the absence of monocyte counting, SII showed a weaker evaluation effect than that of MHR and SIRI, confirming our speculation about the role of monocytes in representing the immune status. For these reasons, we failed to observe a significant association between NLR and various metabolic diseases in our study.

Metabolic dysfunction and diseases are recognized as crucial risk factors for CVD (3, 44); thus, we identified the patients who were exposed to abnormal metabolic status to enable us to provide CVD-related protective interventions timely, reduce the risk of CVD in the future, and lessen the burden of CVD events. Several studies have confirmed a significant correlation of these inflammatory indicators in specific populations for certain specific CVD events, such as myocardial infarction and heart failure (45–47). Some cohort studies have revealed that long-term chronic inflammation could significantly increase the risk of adverse prognosis (48, 49). Our study focused on the natural population and demonstrated that MHR and SIRI had an evaluation effect on 10-year CVD risk, regardless of whether the risk was over 10%/11%, 20%/22%, or 30%, further supporting the previous conclusions. We also noticed that NLR and SII did not have an association with the 10-year CVD risk, and these results were in contrast with some previous studies in which these two inflammatory indicators also showed a good evaluation of the risk of CVD events (45, 47). Except for the fact that these two indicators had subject characteristic preferences and disease adaptation, which was led by the monocyte-mediated immune system, as we mentioned before, our study performed FRS to describe the risk of 10-year CVD, leading to the risk for 10-year CVD risk we derived based on the risk probability, whereas other studies defined various CVD events directly as the outcomes in their regression models. Additionally, the differences in the abovementioned aspects might lead to different results on the relationship between these two indicators and CVD risk in different studies.

The present study had some strengths. Our study first examined inflammation and metabolic abnormalities at the individual level, filling the gap in epidemiological research in this area of inflammation and metabolism. This study provided a detailed and comprehensive classification of metabolic diseases, which could accurately reflect the alteration of metabolic status. We measured MHR, NLR, SII, and SIRI, which used common peripheral blood counts and incorporated immunological effects rather than the traditional inflammatory cytokines, to reflect the inflammatory status. Last, we further assessed 10-year CVD risk by FRS based on metabolic diseases to refine the conclusions of previous studies. We also had some limitations. First, the present study was cross-sectional research, having a limitation in the causal description of inflammation and metabolic diseases. In addition, we did not perform further screening to identify the reason for inflammation and simply excluded the subjects with extremely elevated WBC counts due to recent infections, because of which we failed to clarify the primary reason for the hyper-inflammatory status. Additionally, the present regression models did not contain the consumption of various medicines such as anti-hypertension drugs, and we did not consider the effects of drug administration on inflammation and metabolism levels; the present study was conducted in the natural population and tried to screen and figure out the high-risk population from the natural population whether they used drugs or not, thus we believed our conclusions were still acceptable. Finally, our study population was a natural population, but it still displayed a high prevalence of metabolic abnormalities; thus, it might have led our results to selection bias. In the following study, we will refine the questionnaire and further conduct propensity score matching to eliminate these confounding factors and strengthen the conclusion of the present study.

In conclusion, we screened MHR and SIRI, which had a significant association with MetD and its components, such as MetS, dyslipidemia, and central obesity, in which a higher inflammatory status tended to accompany a higher risk of metabolic diseases. Moreover, we confirmed that the increment of these two indicators could cause a gradually evaluated risk of 10-year CVD. Lastly, by comparing the evaluation effects of these four indicators, we believe it was the immune-inflammation system that was involved in the alteration process of metabolic status.

## Data availability statement

The raw data of the present study will be made available after evaluation and permission by the subject principals. Requests to access the data should be directed to YH (37931208@qq.com) and YS (yxsun@cmu.edu.cn).

## Ethics statement

The studies involving human participants were reviewed and approved by The Ethics Committee of China Medical University (Shenyang, China, ethical approved project identification code: AF-SOP-07-1, 0-01). The patients/participants provided their written informed consent to participate in this study.

## Author contributions

All co-authors participated in the primary research. Conceptualization: YH and YS; project administration: YS, ZL, and XG; methodology: XG and PW; investigation: YZ and SY; writing (original draft preparation): PW; writing (review and editing): YH and PW. All authors contributed to the article and approved the submitted version. 

## Funding

This research was supported by the following fundings: Liaoning Association for Science and Technology (project grant #LNKX2021FH15); the National Key Research and Development Program from the Ministry of Science and Technology of China (project grant #2018YFC1312400, subproject grant #2018YFC1312403); and the Science and Technology Program of Liaoning Province, China (grant #2020JH1/10300002).

## Acknowledgments

We thank cardiologists and the staff from CDCs of Fuxin City, Panjin City, and Liaoyang City in Liaoning Province, who worked hard to ensure the reliability and accuracy of data.

## Conflict of interest

The authors declare that the research was conducted in the absence of any commercial or financial relationships that could be construed as a potential conflict of interest.

## Publisher’s note

All claims expressed in this article are solely those of the authors and do not necessarily represent those of their affiliated organizations, or those of the publisher, the editors and the reviewers. Any product that may be evaluated in this article, or claim that may be made by its manufacturer, is not guaranteed or endorsed by the publisher.
